# Facts about the London Hospital

**Published:** 1888-04-14

**Authors:** 


### Facts About the London Hospital.

It is not often we depart from established usage to give prominence to any particular charitable institution. A rule, however, is sometimes more honoured in the breach than in the observance. Every well-conducted hospital may justly claim support from all who have the power to aid; but there are now and again special circumstances, special necessities, and special merits which add such weight and pertinence to the claim as to make it practically irresistible. The London Hospital at the present moment appears to us to be one of these exceptional cases.

The hospital is now making its quinquennial appeal to the public on behalf of the maintenance fund. The meeting held at the Mansion House last Wednesday, under the presidency of the Lord Mayor, at which the Duke of Cambridge and other eminent persons spoke earnest and eloquent words on behalf of the institution, was but the beginning of an effort which we hope will be more abundantly successful than the most sanguine anticipations.

The London Hospital is not merely a hospital at the East End, it is *the hospital of the East End*. It is the largest hospital in England; "and," all who know will add, "the most needed." During the single year, 1887, over one hundred and four thousand patients were attended—more than two thousand a week, or, excluding Sundays, over three hundred a-day.

With such an enormous number of patients it will surprise no one to be informed that the necessary expenditure exceeds £50,000 a-year. The average total cost of each in-patient is £5 4*s.* 9½*d.*; and the average total cost, including food, medicine, nursing, bedding, rates, and every other outlay, is only 3*s.* 8¾*d.* a-day for each in-patient. The approximate average for each individual out-patient from the beginning to the end of his term of treatment is 3*s.* 11¾*d.* It is clear, therefore, that there is no waste—every pound given to the hospital is made to produce the largest and best possible results.

The necessary outlay is over £50,000 per annum—the assured income is £16,480. There is thus a yawning gulf to be filled each year by voluntary subscriptions, which cannot be satisfied with less than £34,000. Thirty-four thousand pounds a-year to be raised by voluntary contributions for one hospital! It seems impossible; but it is annually done. What is more, so well managed has the hospital been in the past, and so firmly grounded is it in the confidence and regard of the public, that unless some altogether unexpected calamity should occur it will steadily continue to be done.

The working-men of East London themselves value the hospital so highly that they contribute more than £2,000 a-year by one fund alone—"The People's Fund." There are no fewer than a million people whose almost sole resource in sickness is this hospital. The work done in the last year exceeded the work ever done in a single year in any British hospital. All accidents and urgent cases are attended to immediately, and no charge is ever made for either attendance or medicine.

In these times *Specialism* is in the ascendant. Specialists are in everybody's mouth. The London Hospital, as well as being a large general hospital, has a special department for every affection known to specialism. It is in fact *a huge combination of special hospitals.* Diseases of the eye, the ear, the throat, the chest; cases of cancer, fistula, obstetric practice, skin disease—each has its department, and each is presided over by its own eminent specialist.

A feature worthy of special note, and one which shows how ready the hospital is to adapt itself to every circumstance which may add to its usefulness, is the existence within its walls of a separate department for members of the Jewish Faith. There are thousands of poor Jews at the East End. The wealthy Israelites, though comparatively numerous, could by no possibility keep pace with the necessities of their poorer brethren if they were compelled to build and equip separate institutions. By making special provision for the poorer Jews, the hospital has gratified every sentiment of humanity in all classes, and secured for itself the deepest sympathy and the warmest support of all the worthiest members of the Hebrew community.

The London Hospital, in addition to all its other distinctions, claims to be *the largest Children's Hospital in London.* Besides this, it has a large medical college, whose students number four or five hundred, and one of the largest and most efficient nurse-training schools in the world. What more is needed to show that this great institution, by its care of the sick and the sorrowing, by its contributions to medical science and skill, by its thorough culture of medical men for all classes of practice and for all parts of her Majesty's dominions, and by its efficient training of skilled nurses for its own and the country's use; what more is needed to show that to the utmost of its ability and opportunity it devotes itself heart and soul to the great work which it is its function to perform? No argument which we could offer could add anything to the eloquence of this plain narration of facts. Any man who desires to do good, in however small a degree, cannot put his money to better use than by aiding the London Hospital.

